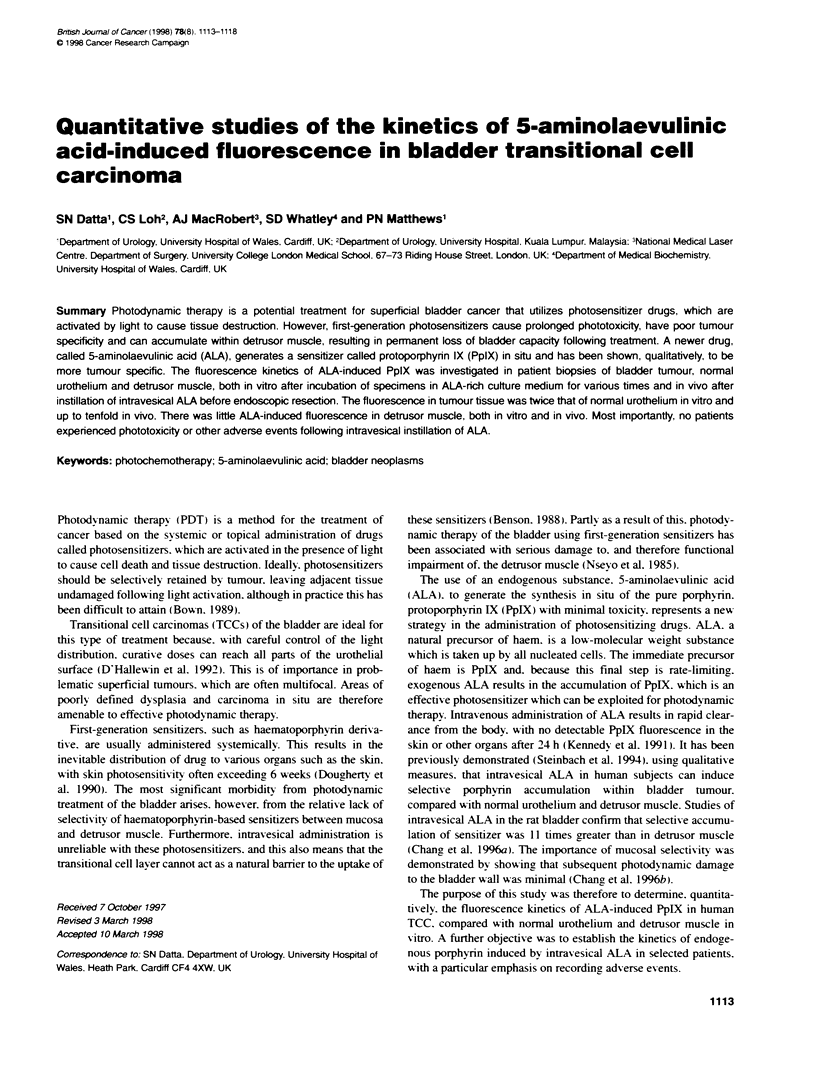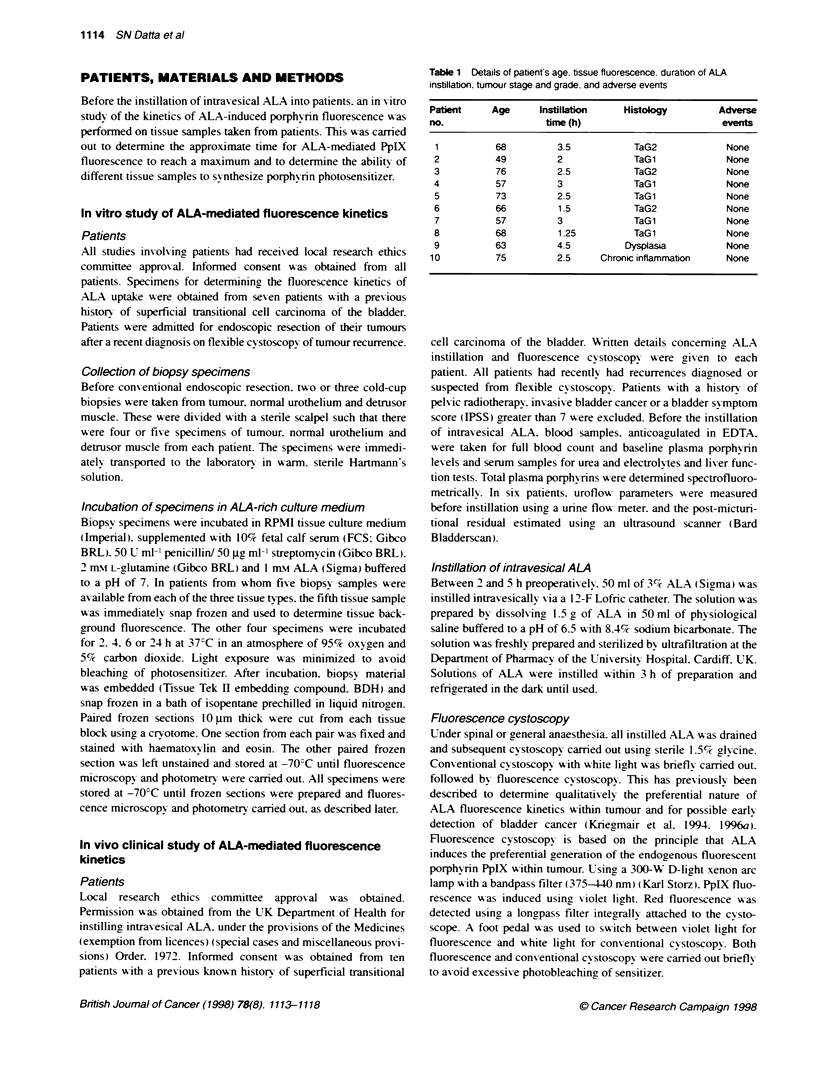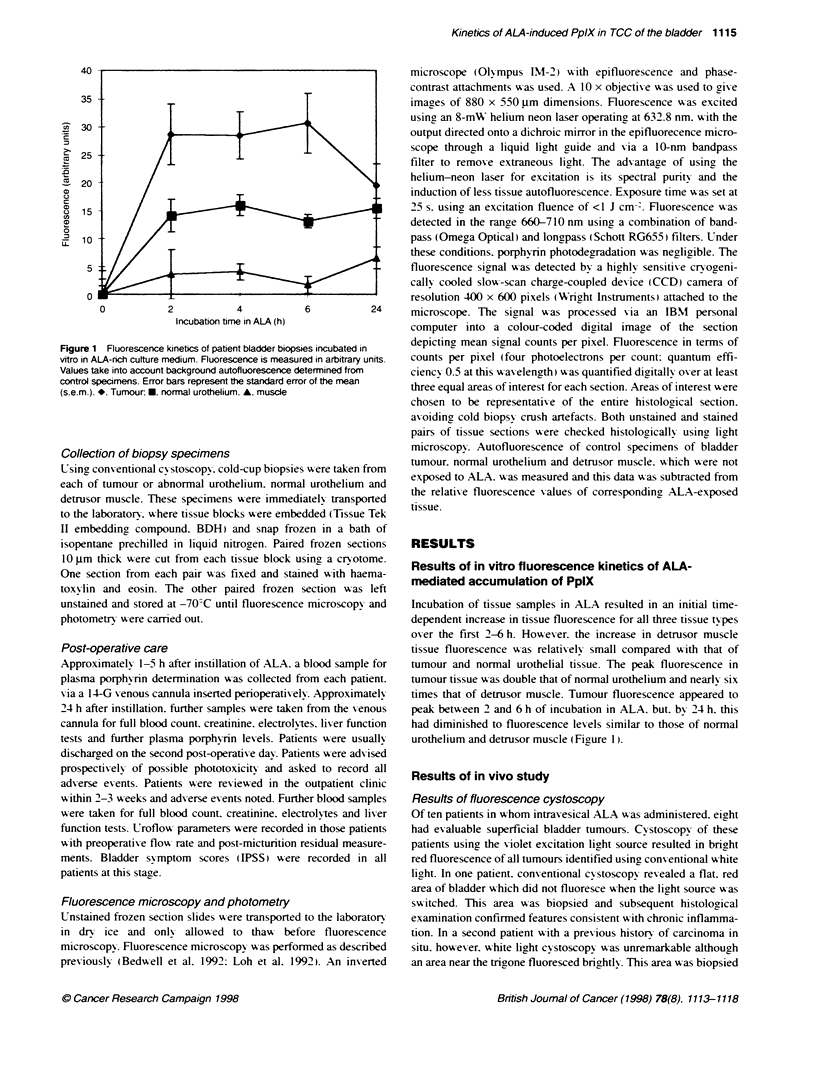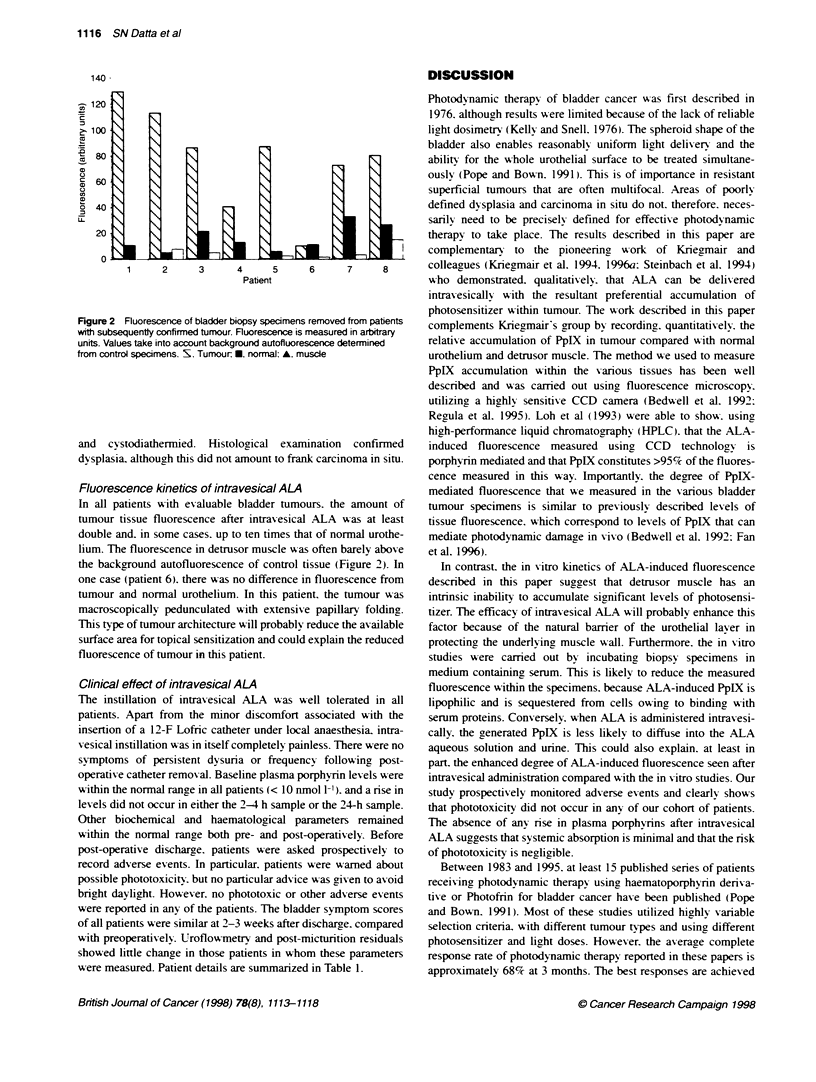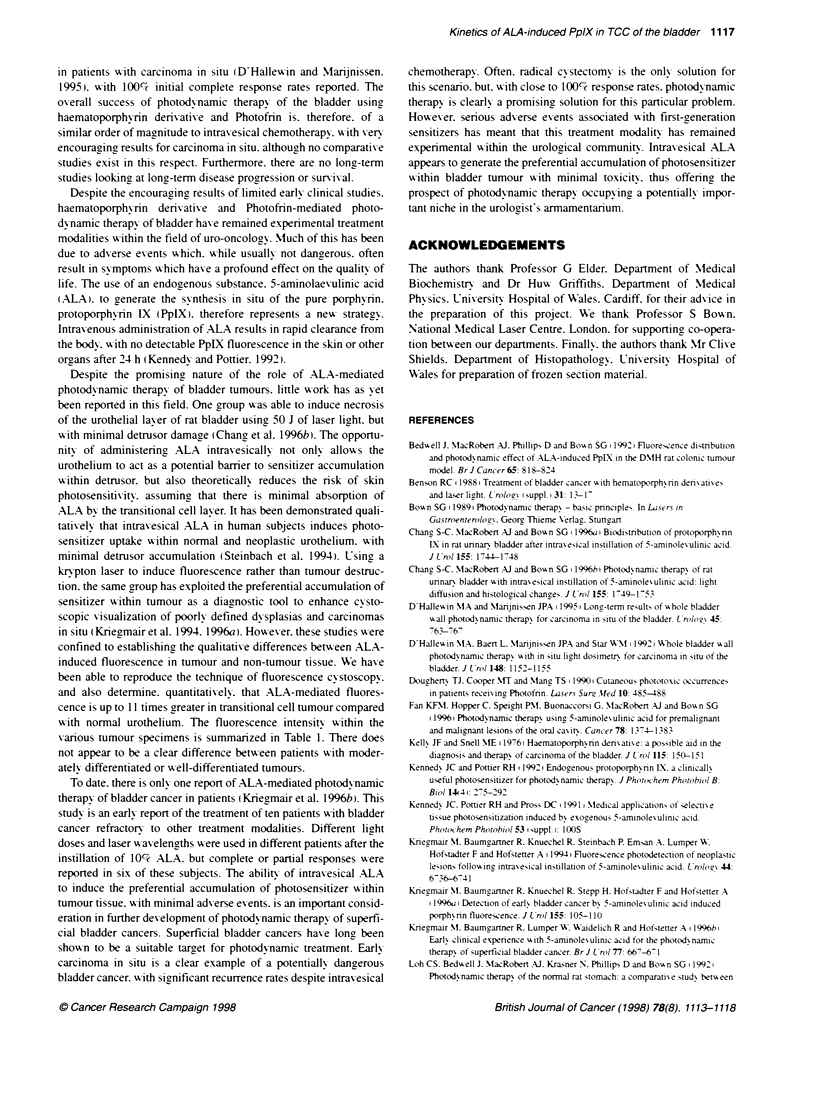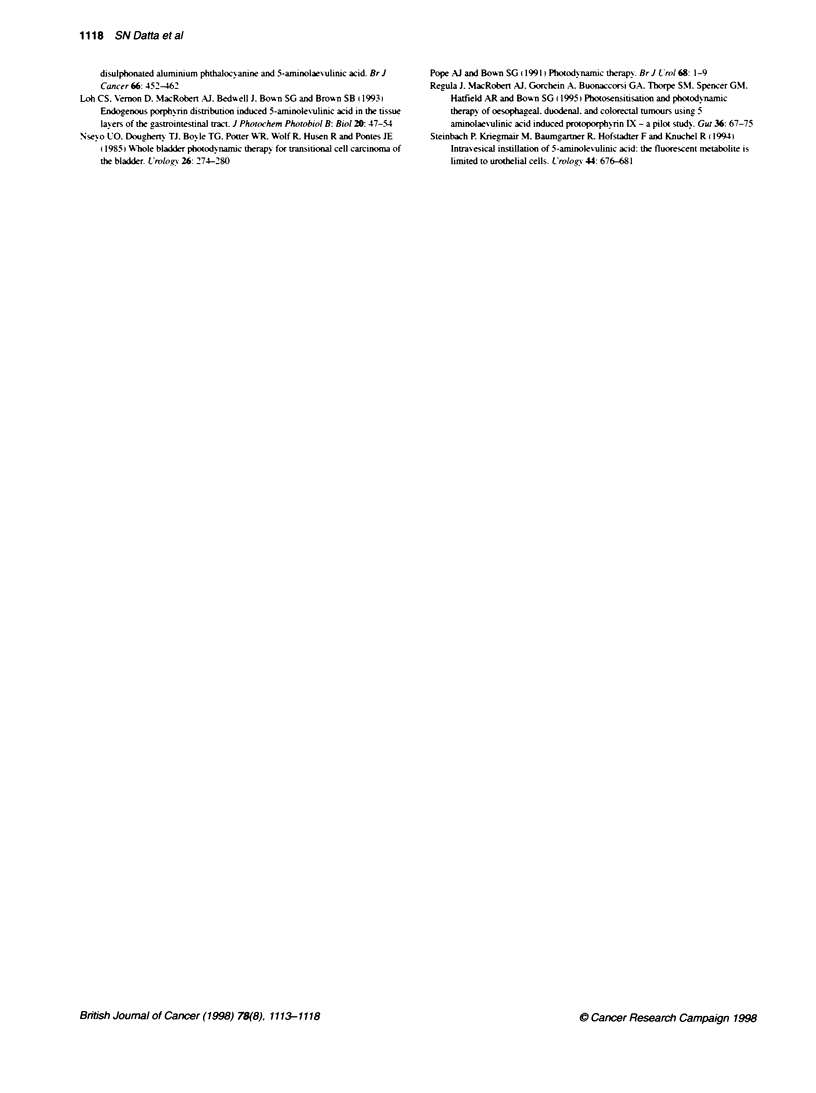# Quantitative studies of the kinetics of 5-aminolaevulinic acid-induced fluorescence in bladder transitional cell carcinoma.

**DOI:** 10.1038/bjc.1998.637

**Published:** 1998-10

**Authors:** S. N. Datta, C. S. Loh, A. J. MacRobert, S. D. Whatley, P. N. Matthews

**Affiliations:** Department of Urology, University Hospital of Wales, Cardiff, UK.

## Abstract

Photodynamic therapy is a potential treatment for superficial bladder cancer that utilizes photosensitizer drugs, which are activated by light to cause tissue destruction. However, first-generation photosensitizers cause prolonged phototoxicity, have poor tumour specificity and can accumulate within detrusor muscle, resulting in permanent loss of bladder capacity following treatment. A newer drug, called 5-aminolaevulinic acid (ALA), generates a sensitizer called protoporphyrin IX (PpIX) in situ and has been shown, qualitatively, to be more tumour specific. The fluorescence kinetics of ALA-induced PpIX was investigated in patient biopsies of bladder tumour, normal urothelium and detrusor muscle, both in vitro after incubation of specimens in ALA-rich culture medium for various times and in vivo after instillation of intravesical ALA before endoscopic resection. The fluorescence in tumour tissue was twice that of normal urothelium in vitro and up to tenfold in vivo. There was little ALA-induced fluorescence in detrusor muscle, both in vitro and in vivo. Most importantly, no patients experienced phototoxicity or other adverse events following intravesical instillation of ALA.


British Journal of Cancer (1998) 78(8), 1113–1118
© 1998 Cancer Research Campaign

### Quantitative studies of the kinetics of 5-aminolaevulinic acid-induced fluorescence in bladder transitional cell carcinoma

**SN Datta[1], CS Loh[2], AJ MacRobert[3], SD Whatley[4] and PN Matthews[1]**

[1]Department of Urology, University Hospital of Wales, Cardiff, UK; [2]Department of Urology, University Hospital, Kuala Lumpur, Malaysia; [3]National Medical Laser Centre, Department of Surgery, University College London Medical School, 67–73 Riding House Street, London, UK; [4]Department of Medical Biochemistry, University Hospital of Wales, Cardiff, UK

**Summary** Photodynamic therapy is a potential treatment for superficial bladder cancer that utilizes photosensitizer drugs, which are activated by light to cause tissue destruction. However, first-generation photosensitizers cause prolonged phototoxicity, have poor tumour specificity and can accumulate within detrusor muscle, resulting in permanent loss of bladder capacity following treatment. A newer drug, called 5-aminolaevulinic acid (ALA), generates a sensitizer called protoporphyrin IX (PpIX) in situ and has been shown, qualitatively, to be more tumour specific. The fluorescence kinetics of ALA-induced PpIX was investigated in patient biopsies of bladder tumour, normal urothelium and detrusor muscle, both in vitro after incubation of specimens in ALA-rich culture medium for various times and in vivo after instillation of intravesical ALA before endoscopic resection. The fluorescence in tumour tissue was twice that of normal urothelium in vitro and up to tenfold in vivo. There was little ALA-induced fluorescence in detrusor muscle, both in vitro and in vivo. Most importantly, no patients experienced phototoxicity or other adverse events following intravesical instillation of ALA.

**Keywords:** photochemotherapy; 5-aminolaevulinic acid; bladder neoplasms

Photodynamic therapy (PDT) is a method for the treatment of cancer based on the systemic or topical administration of drugs called photosensitizers, which are activated in the presence of light to cause cell death and tissue destruction. Ideally, photosensitizers should be selectively retained by tumour, leaving adjacent tissue undamaged following light activation, although in practice this has been difficult to attain (Bown, 1989).

Transitional cell carcinomas (TCCs) of the bladder are ideal for this type of treatment because, with careful control of the light distribution, curative doses can reach all parts of the urothelial surface (D'Hallewin et al, 1992). This is of importance in problematic superficial tumours, which are often multifocal. Areas of poorly defined dysplasia and carcinoma in situ are therefore amenable to effective photodynamic therapy.

First-generation sensitizers, such as haematoporphyrin derivative, are usually administered systemically. This results in the inevitable distribution of drug to various organs such as the skin, with skin photosensitivity often exceeding 6 weeks (Dougherty et al, 1990). The most significant morbidity from photodynamic treatment of the bladder arises, however, from the relative lack of selectivity of haematoporphyrin-based sensitizers between mucosa and detrusor muscle. Furthermore, intravesical administration is unreliable with these photosensitizers, and this also means that the transitional cell layer cannot act as a natural barrier to the uptake of these sensitizers (Benson, 1988). Partly as a result of this, photodynamic therapy of the bladder using first-generation sensitizers has been associated with serious damage to, and therefore functional impairment of, the detrusor muscle (Nseyo et al, 1985).

The use of an endogenous substance, 5-aminolaevulinic acid (ALA), to generate the synthesis in situ of the pure porphyrin, protoporphyrin IX (PpIX) with minimal toxicity, represents a new strategy in the administration of photosensitizing drugs. ALA, a natural precursor of haem, is a low-molecular weight substance which is taken up by all nucleated cells. The immediate precursor of haem is PpIX and, because this final step is rate-limiting, exogenous ALA results in the accumulation of PpIX, which is an effective photosensitizer which can be exploited for photodynamic therapy. Intravenous administration of ALA results in rapid clearance from the body, with no detectable PpIX fluorescence in the skin or other organs after 24 h (Kennedy et al, 1991). It has been previously demonstrated (Steinbach et al, 1994), using qualitative measures, that intravesical ALA in human subjects can induce selective porphyrin accumulation within bladder tumour, compared with normal urothelium and detrusor muscle. Studies of intravesical ALA in the rat bladder confirm that selective accumulation of sensitizer was 11 times greater than in detrusor muscle (Chang et al, 1996*a*). The importance of mucosal selectivity was demonstrated by showing that subsequent photodynamic damage to the bladder wall was minimal (Chang et al, 1996*b*).

The purpose of this study was therefore to determine, quantitatively, the fluorescence kinetics of ALA-induced PpIX in human TCC, compared with normal urothelium and detrusor muscle in vitro. A further objective was to establish the kinetics of endogenous porphyrin induced by intravesical ALA in selected patients, with a particular emphasis on recording adverse events.

*Received 7 October 1997*
*Revised 3 March 1998*
*Accepted 10 March 1998*

*Correspondence to:* SN Datta, Department of Urology, University Hospital of Wales, Heath Park, Cardiff CF4 4XW, UK

### PATIENTS, MATERIALS AND METHODS

Before the instillation of intravesical ALA into patients, an in vitro study of the kinetics of ALA-induced porphyrin fluorescence was performed on tissue samples taken from patients. This was carried out to determine the approximate time for ALA-mediated PpIX fluorescence to reach a maximum and to determine the ability of different tissue samples to synthesize porphyrin photosensitizer.

### In vitro study of ALA-mediated fluorescence kinetics

#### *Patients*

All studies involving patients had received local research ethics committee approval. Informed consent was obtained from all patients. Specimens for determining the fluorescence kinetics of ALA uptake were obtained from seven patients with a previous history of superficial transitional cell carcinoma of the bladder. Patients were admitted for endoscopic resection of their tumours after a recent diagnosis on flexible cystoscopy of tumour recurrence.

#### *Collection of biopsy specimens*

Before conventional endoscopic resection, two or three cold-cup biopsies were taken from tumour, normal urothelium and detrusor muscle. These were divided with a sterile scalpel such that there were four or five specimens of tumour, normal urothelium and detrusor muscle from each patient. The specimens were immediately transported to the laboratory in warm, sterile Hartmann's solution.

#### *Incubation of specimens in ALA-rich culture medium*

Biopsy specimens were incubated in RPMI tissue culture medium (Imperial), supplemented with 10% fetal calf serum (FCS; Gibco BRL), 50 U $ml^{-1}$ penicillin/ 50 μg $ml^{-1}$ streptomycin (Gibco BRL), 2 mM L-glutamine (Gibco BRL) and 1 mM ALA (Sigma) buffered to a pH of 7. In patients from whom five biopsy samples were available from each of the three tissue types, the fifth tissue sample was immediately snap frozen and used to determine tissue background fluorescence. The other four specimens were incubated for 2, 4, 6 or 24 h at 37°C in an atmosphere of 95% oxygen and 5% carbon dioxide. Light exposure was minimized to avoid bleaching of photosensitizer. After incubation, biopsy material was embedded (Tissue Tek II embedding compound, BDH) and snap frozen in a bath of isopentane prechilled in liquid nitrogen. Paired frozen sections 10 μm thick were cut from each tissue block using a cryotome. One section from each pair was fixed and stained with haematoxylin and eosin. The other paired frozen section was left unstained and stored at –70°C until fluorescence microscopy and photometry were carried out. All specimens were stored at –70°C until frozen sections were prepared and fluorescence microscopy and photometry carried out, as described later.

### In vivo clinical study of ALA-mediated fluorescence kinetics

#### *Patients*

Local research ethics committee approval was obtained. Permission was obtained from the UK Department of Health for instilling intravesical ALA, under the provisions of the Medicines (exemption from licences) (special cases and miscellaneous provisions) Order, 1972. Informed consent was obtained from ten patients with a previous known history of superficial transitional cell carcinoma of the bladder. Written details concerning ALA instillation and fluorescence cystoscopy were given to each patient. All patients had recently had recurrences diagnosed or suspected from flexible cystoscopy. Patients with a history of pelvic radiotherapy, invasive bladder cancer or a bladder symptom score (IPSS) greater than 7 were excluded. Before the instillation of intravesical ALA, blood samples, anticoagulated in EDTA, were taken for full blood count and baseline plasma porphyrin levels and serum samples for urea and electrolytes and liver function tests. Total plasma porphyrins were determined spectrofluorometrically. In six patients, uroflow parameters were measured before instillation using a urine flow meter, and the post-micturitional residual estimated using an ultrasound scanner (Bard Bladderscan).

**Table 1** Details of patient's age, tissue fluorescence, duration of ALA instillation, tumour stage and grade, and adverse events

| Patient no. | Age | Instillation time (h) | Histology | Adverse events |
|---|---|---|---|---|
| 1 | 68 | 3.5 | TaG2 | None |
| 2 | 49 | 2 | TaG1 | None |
| 3 | 76 | 2.5 | TaG2 | None |
| 4 | 57 | 3 | TaG1 | None |
| 5 | 73 | 2.5 | TaG1 | None |
| 6 | 66 | 1.5 | TaG2 | None |
| 7 | 57 | 3 | TaG1 | None |
| 8 | 68 | 1.25 | TaG1 | None |
| 9 | 63 | 4.5 | Dysplasia | None |
| 10 | 75 | 2.5 | Chronic inflammation | None |

#### *Instillation of intravesical ALA*

Between 2 and 5 h preoperatively, 50 ml of 3% ALA (Sigma) was instilled intravesically via a 12-F Lofric catheter. The solution was prepared by dissolving 1.5 g of ALA in 50 ml of physiological saline buffered to a pH of 6.5 with 8.4% sodium bicarbonate. The solution was freshly prepared and sterilized by ultrafiltration at the Department of Pharmacy of the University Hospital, Cardiff, UK. Solutions of ALA were instilled within 3 h of preparation and refrigerated in the dark until used.

#### *Fluorescence cystoscopy*

Under spinal or general anaesthesia, all instilled ALA was drained and subsequent cystoscopy carried out using sterile 1.5% glycine. Conventional cystoscopy with white light was briefly carried out, followed by fluorescence cystoscopy. This has previously been described to determine qualitatively the preferential nature of ALA fluorescence kinetics within tumour and for possible early detection of bladder cancer (Kriegmair et al, 1994, 1996*a*). Fluorescence cystoscopy is based on the principle that ALA induces the preferential generation of the endogenous fluorescent porphyrin PpIX within tumour. Using a 300-W D-light xenon arc lamp with a bandpass filter (375–440 nm) (Karl Storz), PpIX fluorescence was induced using violet light. Red fluorescence was detected using a longpass filter integrally attached to the cystoscope. A foot pedal was used to switch between violet light for fluorescence and white light for conventional cystoscopy. Both fluorescence and conventional cystoscopy were carried out briefly to avoid excessive photobleaching of sensitizer.

© Cancer Research Campaign 1998

**Figure 1** Fluorescence kinetics of patient bladder biopsies incubated in vitro in ALA-rich culture medium. Fluorescence is measured in arbitrary units. Values take into account background autofluorescence determined from control specimens. Error bars represent the standard error of the mean (s.e.m.). ◆, Tumour; ■, normal urothelium; ▲, muscle

*Collection of biopsy specimens*

Using conventional cystoscopy, cold-cup biopsies were taken from each of tumour or abnormal urothelium, normal urothelium and detrusor muscle. These specimens were immediately transported to the laboratory, where tissue blocks were embedded (Tissue Tek II embedding compound, BDH) and snap frozen in a bath of isopentane prechilled in liquid nitrogen. Paired frozen sections 10 µm thick were cut from each tissue block using a cryotome. One section from each pair was fixed and stained with haematoxylin and eosin. The other paired frozen section was left unstained and stored at −70°C until fluorescence microscopy and photometry were carried out.

*Post-operative care*

Approximately 1–5 h after instillation of ALA, a blood sample for plasma porphyrin determination was collected from each patient, via a 14-G venous cannula inserted perioperatively. Approximately 24 h after instillation, further samples were taken from the venous cannula for full blood count, creatinine, electrolytes, liver function tests and further plasma porphyrin levels. Patients were usually discharged on the second post-operative day. Patients were advised prospectively of possible phototoxicity and asked to record all adverse events. Patients were reviewed in the outpatient clinic within 2–3 weeks and adverse events noted. Further blood samples were taken for full blood count, creatinine, electrolytes and liver function tests. Uroflow parameters were recorded in those patients with preoperative flow rate and post-micturition residual measurements. Bladder symptom scores (IPSS) were recorded in all patients at this stage.

*Fluorescence microscopy and photometry*

Unstained frozen section slides were transported to the laboratory in dry ice and only allowed to thaw before fluorescence microscopy. Fluorescence microscopy was performed as described previously (Bedwell et al, 1992; Loh et al, 1992). An inverted microscope (Olympus IM-2) with epifluorescence and phase-contrast attachments was used. A 10 × objective was used to give images of 880 × 550 µm dimensions. Fluorescence was excited using an 8-mW helium neon laser operating at 632.8 nm, with the output directed onto a dichroic mirror in the epifluorecence microscope through a liquid light guide and via a 10-nm bandpass filter to remove extraneous light. The advantage of using the helium–neon laser for excitation is its spectral purity and the induction of less tissue autofluorescence. Exposure time was set at 25 s, using an excitation fluence of <1 J $cm^{-2}$. Fluorescence was detected in the range 660–710 nm using a combination of bandpass (Omega Optical) and longpass (Schott RG655) filters. Under these conditions, porphyrin photodegradation was negligible. The fluorescence signal was detected by a highly sensitive cryogenically cooled slow-scan charge-coupled device (CCD) camera of resolution 400 × 600 pixels (Wright Instruments) attached to the microscope. The signal was processed via an IBM personal computer into a colour-coded digital image of the section depicting mean signal counts per pixel. Fluorescence in terms of counts per pixel (four photoelectrons per count; quantum efficiency 0.5 at this wavelength) was quantified digitally over at least three equal areas of interest for each section. Areas of interest were chosen to be representative of the entire histological section, avoiding cold biopsy crush artefacts. Both unstained and stained pairs of tissue sections were checked histologically using light microscopy. Autofluorescence of control specimens of bladder tumour, normal urothelium and detrusor muscle, which were not exposed to ALA, was measured and this data was subtracted from the relative fluorescence values of corresponding ALA-exposed tissue.

### RESULTS

### Results of in vitro fluorescence kinetics of ALA-mediated accumulation of PpIX

Incubation of tissue samples in ALA resulted in an initial time-dependent increase in tissue fluorescence for all three tissue types over the first 2–6 h. However, the increase in detrusor muscle tissue fluorescence was relatively small compared with that of tumour and normal urothelial tissue. The peak fluorescence in tumour tissue was double that of normal urothelium and nearly six times that of detrusor muscle. Tumour fluorescence appeared to peak between 2 and 6 h of incubation in ALA, but, by 24 h, this had diminished to fluorescence levels similar to those of normal urothelium and detrusor muscle (Figure 1).

### Results of in vivo study

*Results of fluorescence cystoscopy*

Of ten patients in whom intravesical ALA was administered, eight had evaluable superficial bladder tumours. Cystoscopy of these patients using the violet excitation light source resulted in bright red fluorescence of all tumours identified using conventional white light. In one patient, conventional cystoscopy revealed a flat, red area of bladder which did not fluoresce when the light source was switched. This area was biopsied and subsequent histological examination confirmed features consistent with chronic inflammation. In a second patient with a previous history of carcinoma in situ, however, white light cystoscopy was unremarkable although an area near the trigone fluoresced brightly. This area was biopsied

**Figure 2** Fluorescence of bladder biopsy specimens removed from patients with subsequently confirmed tumour. Fluorescence is measured in arbitrary units. Values take into account background autofluorescence determined from control specimens. ⧅, Tumour; ■, normal; ▲, muscle and cystodiathermied. Histological examination confirmed dysplasia, although this did not amount to frank carcinoma in situ.

### *Fluorescence kinetics of intravesical ALA*

In all patients with evaluable bladder tumours, the amount of tumour tissue fluorescence after intravesical ALA was at least double and, in some cases, up to ten times that of normal urothelium. The fluorescence in detrusor muscle was often barely above the background autofluorescence of control tissue (Figure 2). In one case (patient 6), there was no difference in fluorescence from tumour and normal urothelium. In this patient, the tumour was macroscopically pedunculated with extensive papillary folding. This type of tumour architecture will probably reduce the available surface area for topical sensitization and could explain the reduced fluorescence of tumour in this patient.

### *Clinical effect of intravesical ALA*

The instillation of intravesical ALA was well tolerated in all patients. Apart from the minor discomfort associated with the insertion of a 12-F Lofric catheter under local anaesthesia, intravesical instillation was in itself completely painless. There were no symptoms of persistent dysuria or frequency following post-operative catheter removal. Baseline plasma porphyrin levels were within the normal range in all patients (< 10 nmol $l^{-1}$), and a rise in levels did not occur in either the 2–4 h sample or the 24-h sample. Other biochemical and haematological parameters remained within the normal range both pre- and post-operatively. Before post-operative discharge, patients were asked prospectively to record adverse events. In particular, patients were warned about possible phototoxicity, but no particular advice was given to avoid bright daylight. However, no phototoxic or other adverse events were reported in any of the patients. The bladder symptom scores of all patients were similar at 2–3 weeks after discharge, compared with preoperatively. Uroflowmetry and post-micturition residuals showed little change in those patients in whom these parameters were measured. Patient details are summarized in Table 1.

### DISCUSSION

Photodynamic therapy of bladder cancer was first described in 1976, although results were limited because of the lack of reliable light dosimetry (Kelly and Snell, 1976). The spheroid shape of the bladder also enables reasonably uniform light delivery and the ability for the whole urothelial surface to be treated simultaneously (Pope and Bown, 1991). This is of importance in resistant superficial tumours that are often multifocal. Areas of poorly defined dysplasia and carcinoma in situ do not, therefore, necessarily need to be precisely defined for effective photodynamic therapy to take place. The results described in this paper are complementary to the pioneering work of Kriegmair and colleagues (Kriegmair et al, 1994, 1996*a*; Steinbach et al, 1994) who demonstrated, qualitatively, that ALA can be delivered intravesically with the resultant preferential accumulation of photosensitizer within tumour. The work described in this paper complements Kriegmair's group by recording, quantitatively, the relative accumulation of PpIX in tumour compared with normal urothelium and detrusor muscle. The method we used to measure PpIX accumulation within the various tissues has been well described and was carried out using fluorescence microscopy, utilizing a highly sensitive CCD camera (Bedwell et al, 1992; Regula et al, 1995). Loh et al (1993) were able to show, using high-performance liquid chromatography (HPLC), that the ALA-induced fluorescence measured using CCD technology is porphyrin mediated and that PpIX constitutes >95% of the fluorescence measured in this way. Importantly, the degree of PpIX-mediated fluorescence that we measured in the various bladder tumour specimens is similar to previously described levels of tissue fluorescence, which correspond to levels of PpIX that can mediate photodynamic damage in vivo (Bedwell et al, 1992; Fan et al, 1996).

In contrast, the in vitro kinetics of ALA-induced fluorescence described in this paper suggest that detrusor muscle has an intrinsic inability to accumulate significant levels of photosensitizer. The efficacy of intravesical ALA will probably enhance this factor because of the natural barrier of the urothelial layer in protecting the underlying muscle wall. Furthermore, the in vitro studies were carried out by incubating biopsy specimens in medium containing serum. This is likely to reduce the measured fluorescence within the specimens, because ALA-induced PpIX is lipophilic and is sequestered from cells owing to binding with serum proteins. Conversely, when ALA is administered intravesically, the generated PpIX is less likely to diffuse into the ALA aqueous solution and urine. This could also explain, at least in part, the enhanced degree of ALA-induced fluorescence seen after intravesical administration compared with the in vitro studies. Our study prospectively monitored adverse events and clearly shows that phototoxicity did not occur in any of our cohort of patients. The absence of any rise in plasma porphyrins after intravesical ALA suggests that systemic absorption is minimal and that the risk of phototoxicity is negligible.

Between 1983 and 1995, at least 15 published series of patients receiving photodynamic therapy using haematoporphyrin derivative or Photofrin for bladder cancer have been published (Pope and Bown, 1991). Most of these studies utilized highly variable selection criteria, with different tumour types and using different photosensitizer and light doses. However, the average complete response rate of photodynamic therapy reported in these papers is approximately 68% at 3 months. The best responses are achieved

© Cancer Research Campaign 1998

in patients with carcinoma in situ (D'Hallewin and Marijnissen, 1995), with 100% initial complete response rates reported. The overall success of photodynamic therapy of the bladder using haematoporphyrin derivative and Photofrin is, therefore, of a similar order of magnitude to intravesical chemotherapy, with very encouraging results for carcinoma in situ, although no comparative studies exist in this respect. Furthermore, there are no long-term studies looking at long-term disease progression or survival.

Despite the encouraging results of limited early clinical studies, haematoporphyrin derivative and Photofrin-mediated photodynamic therapy of bladder have remained experimental treatment modalities within the field of uro-oncology. Much of this has been due to adverse events which, while usually not dangerous, often result in symptoms which have a profound effect on the quality of life. The use of an endogenous substance, 5-aminolaevulinic acid (ALA), to generate the synthesis in situ of the pure porphyrin, protoporphyrin IX (PpIX), therefore represents a new strategy. Intravenous administration of ALA results in rapid clearance from the body, with no detectable PpIX fluorescence in the skin or other organs after 24 h (Kennedy and Pottier, 1992).

Despite the promising nature of the role of ALA-mediated photodynamic therapy of bladder tumours, little work has as yet been reported in this field. One group was able to induce necrosis of the urothelial layer of rat bladder using 50 J of laser light, but with minimal detrusor damage (Chang et al, 1996*b*). The opportunity of administering ALA intravesically not only allows the urothelium to act as a potential barrier to sensitizer accumulation within detrusor, but also theoretically reduces the risk of skin photosensitivity, assuming that there is minimal absorption of ALA by the transitional cell layer. It has been demonstrated qualitatively that intravesical ALA in human subjects induces photosensitizer uptake within normal and neoplastic urothelium, with minimal detrusor accumulation (Steinbach et al, 1994). Using a krypton laser to induce fluorescence rather than tumour destruction, the same group has exploited the preferential accumulation of sensitizer within tumour as a diagnostic tool to enhance cystoscopic visualization of poorly defined dysplasias and carcinomas in situ (Kriegmair et al, 1994, 1996*a*). However, these studies were confined to establishing the qualitative differences between ALA-induced fluorescence in tumour and non-tumour tissue. We have been able to reproduce the technique of fluorescence cystoscopy, and also determine, quantitatively, that ALA-mediated fluorescence is up to 11 times greater in transitional cell tumour compared with normal urothelium. The fluorescence intensity within the various tumour specimens is summarized in Table 1. There does not appear to be a clear difference between patients with moderately differentiated or well-differentiated tumours.

To date, there is only one report of ALA-mediated photodynamic therapy of bladder cancer in patients (Kriegmair et al, 1996*b*). This study is an early report of the treatment of ten patients with bladder cancer refractory to other treatment modalities. Different light doses and laser wavelengths were used in different patients after the instillation of 10% ALA, but complete or partial responses were reported in six of these subjects. The ability of intravesical ALA to induce the preferential accumulation of photosensitizer within tumour tissue, with minimal adverse events, is an important consideration in further development of photodynamic therapy of superficial bladder cancers. Superficial bladder cancers have long been shown to be a suitable target for photodynamic treatment. Early carcinoma in situ is a clear example of a potentially dangerous bladder cancer, with significant recurrence rates despite intravesical chemotherapy. Often, radical cystectomy is the only solution for this scenario, but, with close to 100% response rates, photodynamic therapy is clearly a promising solution for this particular problem. However, serious adverse events associated with first-generation sensitizers has meant that this treatment modality has remained experimental within the urological community. Intravesical ALA appears to generate the preferential accumulation of photosensitizer within bladder tumour with minimal toxicity, thus offering the prospect of photodynamic therapy occupying a potentially important niche in the urologist's armamentarium.

### ACKNOWLEDGEMENTS

The authors thank Professor G Elder, Department of Medical Biochemistry and Dr Huw Griffiths, Department of Medical Physics, University Hospital of Wales, Cardiff, for their advice in the preparation of this project. We thank Professor S Bown, National Medical Laser Centre, London, for supporting co-operation between our departments. Finally, the authors thank Mr Clive Shields, Department of Histopathology, University Hospital of Wales for preparation of frozen section material.

### REFERENCES

Bedwell J, MacRobert AJ, Phillips D and Bown SG (1992) Fluorescence distribution and photodynamic effect of ALA-induced PpIX in the DMH rat colonic tumour model. *Br J Cancer* **65**: 818–824

Benson RC (1988) Treatment of bladder cancer with hematoporphyrin derivatives and laser light. *Urology* (suppl.) **31**: 13–17

Bown SG (1989) Photodynamic therapy – basic principles. In *Lasers in Gastroenterology*. Georg Thieme Verlag, Stuttgart

Chang S-C, MacRobert AJ and Bown SG (1996*a*) Biodistribution of protoporphyrin IX in rat urinary bladder after intravesical instillation of 5-aminolevulinic acid. *J Urol* **155**: 1744–1748

Chang S-C, MacRobert AJ and Bown SG (1996*b*) Photodynamic therapy of rat urinary bladder with intravesical instillation of 5-aminolevulinic acid: light diffusion and histological changes. *J Urol* **155**: 1749–1753

D'Hallewin MA and Marijnissen JPA (1995) Long-term results of whole bladder wall photodynamic therapy for carcinoma in situ of the bladder. *Urology* **45**: 763–767

D'Hallewin MA, Baert L, Marijnissen JPA and Star WM (1992) Whole bladder wall photodynamic therapy with in situ light dosimetry for carcinoma in situ of the bladder. *J Urol* **148**: 1152–1155

Dougherty TJ, Cooper MT and Mang TS (1990) Cutaneous phototoxic occurrences in patients receiving Photofrin. *Lasers Surg Med* **10**: 485–488

Fan KFM, Hopper C, Speight PM, Buonaccorsi G, MacRobert AJ and Bown SG (1996) Photodynamic therapy using 5-aminolevulinic acid for premalignant and malignant lesions of the oral cavity. *Cancer* **78**: 1374–1383

Kelly JF and Snell ME (1976) Haematoporphyrin derivative: a possible aid in the diagnosis and therapy of carcinoma of the bladder. *J Urol* **115**: 150–151

Kennedy JC and Pottier RH (1992) Endogenous protoporphyrin IX, a clinically useful photosensitizer for photodynamic therapy. *J Photochem Photobiol B: Biol* **14**(4): 275–292

Kennedy JC, Pottier RH and Pross DC (1991) Medical applications of selective tissue photosensitization induced by exogenous 5-aminolevulinic acid. *Photochem Photobiol* **53** (suppl.): 100S

Kriegmair M, Baumgartner R, Knuechel R, Steinbach P, Emsan A, Lumper W, Hofstadter F and Hofstetter A (1994) Fluorescence photodetection of neoplastic lesions following intravesical instillation of 5-aminolevulinic acid. *Urology* **44**: 6736–6741

Kriegmair M, Baumgartner R, Knuechel R, Stepp H, Hofstadter F and Hofstetter A (1996*a*) Detection of early bladder cancer by 5-aminolevulinic acid induced porphyrin fluorescence. *J Urol* **155**: 105–110

Kriegmair M, Baumgartner R, Lumper W, Waidelich R and Hofstetter A (1996*b*) Early clinical experience with 5-aminolevulinic acid for the photodynamic therapy of superficial bladder cancer. *Br J Urol* **77**: 667–671

Loh CS, Bedwell J, MacRobert AJ, Krasner N, Phillips D and Bown SG (1992) Photodynamic therapy of the normal rat stomach: a comparative study between disulphonated aluminium phthalocyanine and 5-aminolaevulinic acid. *Br J Cancer* **66**: 452–462
Loh CS, Vernon D, MacRobert AJ, Bedwell J, Bown SG and Brown SB (1993) Endogenous porphyrin distribution induced 5-aminolevulinic acid in the tissue layers of the gastrointestinal tract. *J Photochem Photobiol B: Biol* **20**: 47–54
Nseyo UO, Dougherty TJ, Boyle TG, Potter WR, Wolf R, Husen R and Pontes JE (1985) Whole bladder photodynamic therapy for transitional cell carcinoma of the bladder. *Urology* **26**: 274–280
Pope AJ and Bown SG (1991) Photodynamic therapy. *Br J Urol* **68**: 1–9
Regula J, MacRobert AJ, Gorchein A, Buonaccorsi GA, Thorpe SM, Spencer GM, Hatfield AR and Bown SG (1995) Photosensitisation and photodynamic therapy of oesophageal, duodenal, and colorectal tumours using 5 aminolaevulinic acid induced protoporphyrin IX – a pilot study. *Gut* **36**: 67–75
Steinbach P, Kriegmair M, Baumgartner R, Hofstadter F and Knuchel R (1994) Intravesical instillation of 5-aminolevulinic acid: the fluorescent metabolite is limited to urothelial cells. *Urology* **44**: 676–681

*© Cancer Research Campaign 1998*